# Association of lifestyle modification with the development of cardiovascular disease in gastric cancer patients who underwent gastrectomy: A nationwide population‐based study

**DOI:** 10.1002/cam4.70038

**Published:** 2024-07-24

**Authors:** Bokyung Kim, Kyungdo Han, Soo‐Jeong Cho

**Affiliations:** ^1^ Department of Internal Medicine and Liver Research Institute Seoul National University Hospital, Seoul National University College of Medicine Seoul South Korea; ^2^ Department of Statistics and Actuarial Science Soongsil University Seoul South Korea

**Keywords:** alcohol drinking, cardiovascular diseases, physical activity, smoking, stomach neoplasm

## Abstract

**Background:**

While cancer patients are at an increased risk of cardiovascular disease (CVD), the role of modifiable risk factors remains poorly understood. This study investigated whether lifestyle modifications affect CVD development in gastric cancer patients who undergo surgery.

**Methods:**

Using data from the Korean National Health Insurance Service (NHIS), gastric cancer patients who underwent surgery from 2010 to 2017 were identified. Lifestyle behaviours, surveyed within 2 years before and after surgery were analysed. Incident CVD, defined as a composite of myocardial infarction and stroke, was compared among subgroups of lifestyle behaviour changes.

**Results:**

Among 22,211 gastrectomy patients, 628 (2.8%) developed CVD (5.68/1000 person‐years). Persistent smokers (HR: 1.72, 95% CI: 1.33–2.22) and new smokers (HR: 1.85, 95% CI: 1.04–3.30) faced higher CVD risks than non‐smokers, with an especially pronounced risk in persistent‐smoking females (HR: 3.89, 95% CI: 1.20–12.62). Smoking cessation showed no significant risk difference compared to non‐smokers (HR: 1.16, 95% CI: 0.93–1.43). Female new drinkers had a higher CVD risk than non‐drinking females (HR: 2.89, 95% CI: 1.06–7.88), while men did not show such association. Changes in physical activity, when compared to physical inactivity, were not associated with CVD risk.

**Conclusion:**

Gastric cancer patients who smoked after surgery were more likely to develop CVD irrespective of their prior smoking status, with a notable vulnerability in persistent female smokers. Smoking cessation could potentially mitigate CVD risk to levels observed in non‐smokers. Alcohol intake should be avoided following surgery, especially for female gastric cancer patients.

## Graphical abstract

Gastric cancer patients who smoke after surgery are more likely to develop cardiovascular disease (CVD) irrespective of their prior smoking status, while smoking cessation could potentially mitigate CVD risk to levels observed in non‐smokers. Female gastric cancer patients, who started consuming alcohol after gastrectomy, have higher CVD risk compared to non‐drinking females.

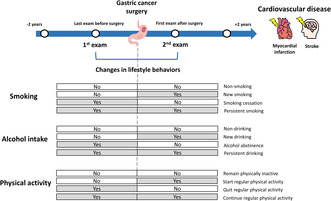

## 1 INTRODUCTION

Cancer is a known risk factor for the development of cardiovascular disease (CVD) and CVD‐related mortality.^1^, ^2^, ^3^, ^4^ In fact, patients with cancer face a 37% higher risk of CVD than the general population.^5^ This elevated risk can be attributed to shared risk factors, such as age, diabetes, hypertension, hyperlipidaemia, obesity and lifestyle factors including smoking, drinking and physical inactivity.^1^, ^6^, ^7^, ^8^ Additionally, tumour cells can produce pro‐inflammatory cytokines and chemokines, which contribute to the development of atherosclerosis.^1^ Certain cancer treatments can also have detrimental effects on the cardiovascular and cerebrovascular systems.^1^, ^9^ Given the substantial risk of CVD in cancer patients, there is a growing concern regarding the need for secondary preventive measures in both clinical and public health settings. For instance, a recent study using the UK Biobank data, reported that a healthy lifestyle was associated with reduced CVD risk among cancer patients.^10^

Gastric cancer is one of the most common cancers worldwide, with over one million new cases reported in 2020.^11^ Gastric cancer patients face a notably elevated risk of CVD‐related mortality.^12^ Given the significant impact of CVD on the prognosis and survival of gastric cancer patients, it is crucial to identify modifiable risk factors that could be targeted for preventing CVD. However, current evidence linking lifestyle factors and CVD development in gastric cancer patients remains sparse. While there have been studies assessing the CVD risk among various cancers based on smoking behaviours, research focusing on gastric cancer patients and the impact of changes in various lifestyle factors including smoking, alcohol intake and physical activity, is limited.^13^, ^14^ Therefore, our study aimed to investigate the effect of lifestyle modifications, including changes in smoking, alcohol intake and regular physical activity, on CVD development in gastric cancer patients undergoing surgery.

## 2 METHODS

### 2.1 Data source

Using claims data from the Korean National Health Insurance Service (NHIS), which offers mandatory health insurance coverage, we conducted a population‐based nationwide cohort study. The NHIS data include medical records, pharmaceutical visits, demographic data and general health examination data. The general health examination data, linked to the National Health Screening database, include blood tests, physical examinations and lifestyle factor questionnaires.^15^, ^16^, ^17^ Biennial health check‐ups are provided to individuals aged ≥40 and all employees regardless of their age, in South Korea. In the NHIS database, diagnoses are recorded by the International Classification of Diseases, 10th revision Clinical Modification (ICD‐10‐CM) codes. In this study, lifestyle factors including smoking, alcohol intake and physical activity were identified by lifestyle factor questionnaires from the general health examination data and the diagnosis of CVD were identified by medical records from NHIS health care utilisation data.

The requirement for written consent was waived by the Institutional Review Board of Seoul National University Hospital (No. 2308‐104‐1459).

### 2.2 Study design and study population

**FIGURE 1:**
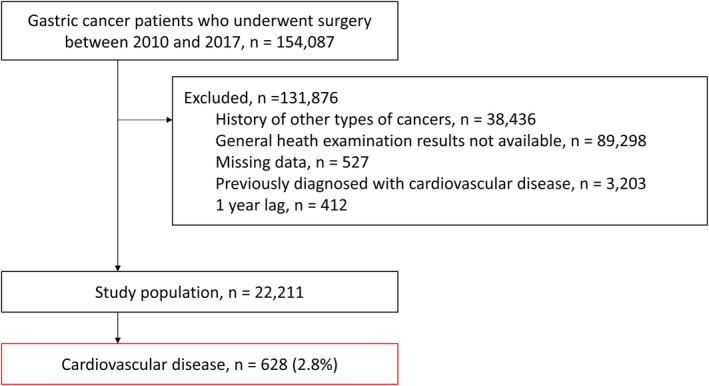
Flowchart of population selection.

**FIGURE 2:**
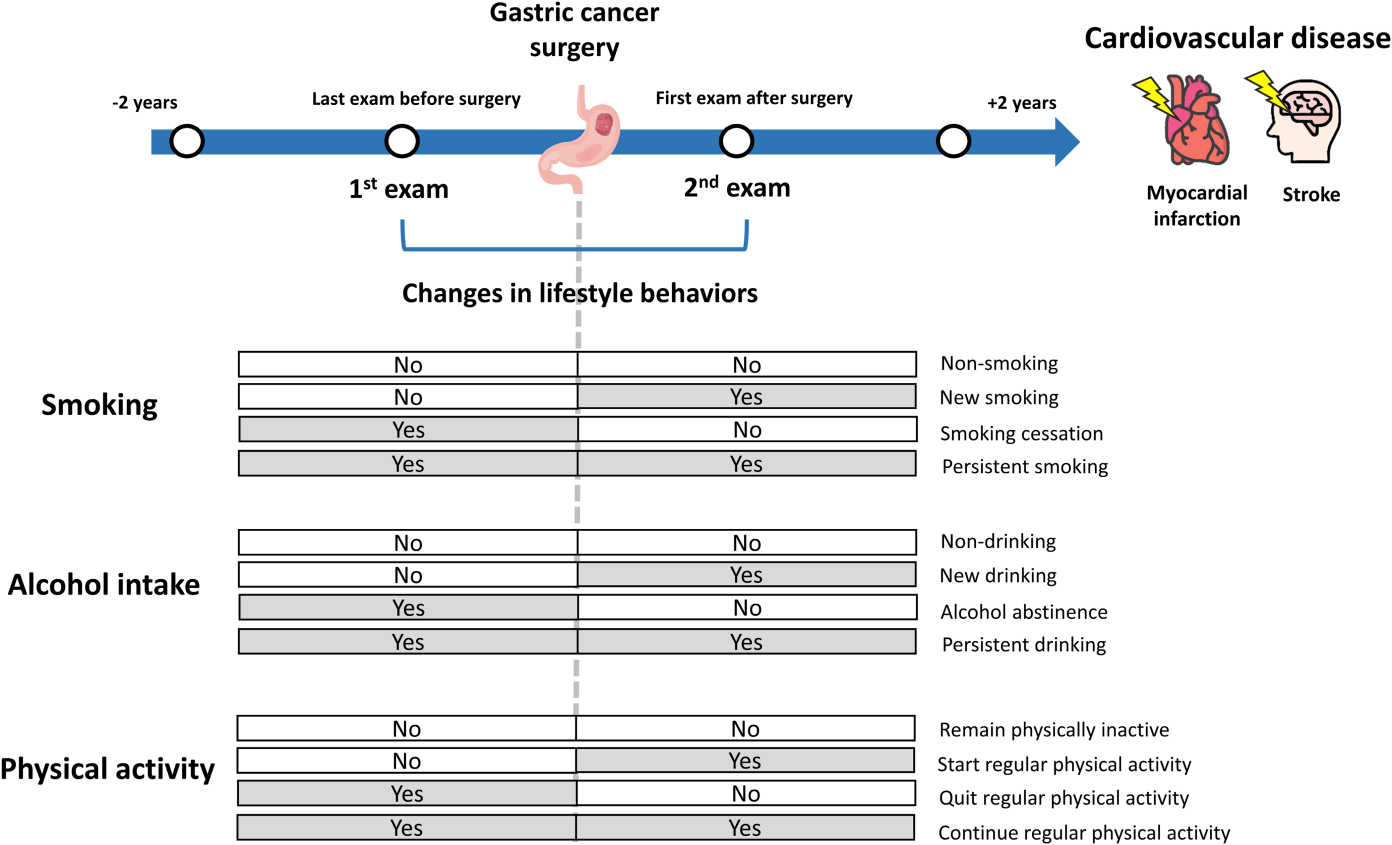
Study design and classification of study population.

The flow of population selection is illustrated in Figure 1 and the study design and classification of the study population are illustrated in Figure 2. Gastric cancer patients aged ≥20 years who underwent surgery between 1 January 2010 and 31 December 2017 were identified. Gastric cancer was defined by diagnostic code for gastric cancer (C16). Patients were included if they had completed at least two general health examinations, one within 2 years before surgery (first exam) and another within 2 years after surgery (second exam). The post‐surgery health examination (second exam) date was defined as the index date. When multiple health check‐up data were available within the 2 years before or after surgery, we used the results from the last exam before surgery and the first exam after surgery to ensure the most relevant data were included and to maintain consistency in our analysis. Patients with missing lifestyle behaviour questionnaire data (either smoking, alcohol intake, or physical activity) and those previously diagnosed with other cancers (C00–C15, C17–C97) or CVD before the index date were excluded. To ensure that CVD was newly diagnosed, we set a one‐year lag period to exclude patients who developed CVD within 1 year after the index date.

### 2.3 Study outcome

The primary outcome of this study was a new diagnosis of CVD, defined as a composite of myocardial infarction (ICD‐10 codes I21 or I22, requiring hospital admission and stroke (ICD‐10 codes I63 or I64, requiring hospital admission and an imaging study, either a brain CT or MRI), as provided by the NHIS claims database. The final follow‐up date was 31 December 2019.

### 2.4 Covariates and lifestyle behaviours

Demographic data, health examination data, comorbidities, type of gastrectomy (total gastrectomy or subtotal gastrectomy) and income status were identified as covariates. The health examination data consisted of lifestyle behaviour surveys, blood pressure measurements, body mass index (BMI) values and blood tests, including fasting blood glucose, glomerular filtration rate (GFR) and lipid profiles. Comorbidities included diabetes mellitus, hypertension, dyslipidaemia, chronic kidney disease and obesity. Table S1 provides detailed definitions of these covariates.

Patients' lifestyle behaviours were identified using self‐administered questionnaires. We assessed their smoking, drinking and physical activity status at the time of each examination. Regular physical activity was defined as moderate physical activity ≥5 times/week or vigorous physical activity ≥3 times/week.^18^

To evaluate changes in lifestyle behaviours, we compared patients' lifestyle behaviours before and after surgery (Figure 2). These changes were classified into (1) smoking: persistent smoking, smoking cessation, new smoking and non‐smoking; (2) alcohol intake: persistent drinking, alcohol abstinence, new drinking and non‐drinking; and (3) physical activity: remaining physically inactive, starting regular physical activity, quitting regular physical activity and continuing regular physical activity.

### 2.5 Statistical analysis

Data are presented as numbers and percentages (categorical variables) and means ± standard deviations (continuous variables). Baseline characteristics were compared using the chi‐square test and *t*‐test. CVD incidence was analysed by dividing the number of new‐onset CVD cases by the total follow‐up period (per 1000 person‐years). We conducted multivariable Cox proportional hazard regression analyses to identify the association between changes in lifestyle behaviours and new‐onset CVD in three models: (1) model 1, unadjusted; (2) model 2, adjusted for age and sex; and (3) model 3, adjusted for age, sex, smoking, drinking, physical activity and comorbidities. To identify possible patient groups that might benefit from lifestyle modification, we performed subgroup analyses based on age, sex and gastrectomy type. A two‐sided *p*‐value of less than 0.05 was considered to be statistically significant. All statistical analyses in this study were performed using SAS version 9.4 (SAS Institute, Cary, NC, USA).

## 3 RESULTS

### 3.1 Study population

Between January 2010 and December 2017, a total of 154,087 patients were diagnosed with gastric cancer and subsequently underwent surgery. Of those, 131,876 were excluded due to a history of other types of cancers (*n* = 38,436), unavailable general health examination data (*n* = 89,298), missing data (*n* = 527), a prior diagnosis of CVD before the index date (*n* = 3203) or a one‐year lag period (*n* = 412). This left 22,211 patients in the study population, of whom 628 (2.8%) newly developed CVD during a cumulative 110,562 person‐years of follow‐up (5.68 per 1000 person‐years) (Figure 1).

Table 1 shows the baseline characteristics of the study population based on the post‐surgery health examination (second exam). Patients who developed CVD were more likely to be older, male, smokers and heavy drinkers than those who did not develop CVD. They also tended to have higher fasting blood glucose levels, lower GFR, higher blood pressure and more comorbidities, including diabetes mellitus, hypertension, dyslipidaemia and chronic kidney disease, compared to patients who did not develop CVD.

**TABLE 1:** Baseline characteristics of subjects.

	Cardiovascular disease^a^		*p*‐value
	No (*n* = 21,583)	Yes (*n* = 628)	
Age			
<40 years	704 (3.26)	6 (0.96)	<0.001
40–64 years	13,991 (64.82)	260 (41.4)	
≥65 years	6888 (31.91)	362 (57.64)	
Sex			
Male	14,897 (69.02)	488 (77.71)	<0.001
Female	6686 (30.98)	140 (22.29)	
Low income (<25%)	2878 (17.34)	431 (18.72)	0.88
Smoking			
Non‐smoker	11,056 (51.23)	310 (49.36)	<0.001
Ex‐smoker	8611 (39.9)	228 (36.31)	
Current smoker	1916 (8.88)	90 (14.33)	
Alcohol intake^b^			
Non	18,568 (86.03)	516 (82.17)	0.02
Mild	2686 (12.44)	97 (15.45)	
Heavy	329 (1.52)	15 (2.39)	
Regular physical activity			
No	15,450 (71.58)	466 (74.20)	0.15
Yes	6133 (28.42)	162 (25.80)	
BMI, mean (SD), kg/m^2^	21.71 (2.77)	21.78 (2.91)	0.53
Fasting blood glucose, mean (SD), mg/dL	100.35 (25.12)	106.32 (29.75)	<0.001
GFR, mean (SD), mL/min/1.73 m^2^	91.93 (24.29)	87.65 (25.19)	<0.001
SBP, mean (SD), mmHg	118.64 (14.64)	123.36 (15.84)	<0.001
DBP, mean (SD), mmHg	73.06 (9.5)	74.12 (9.8)	0.006
Total cholesterol, mean (SD), mg/dL	175.85 (32.66)	175.94 (35.69)	0.95
HDL‐cholesterol, mean (SD), mg/dL	56.94 (15.86)	55.17 (15.16)	0.006
LDL‐cholesterol, mean (SD), mg/dL	98.75 (28.76)	98.5 (31.04)	0.83
TG, mean (SD), mg/dL	90.32 (0.57)	99.3 (3.75)	<0.001
Comorbidities			
Diabetes mellitus	3344 (15.49)	182 (28.98)	<0.001
Hypertension	6009 (27.84)	284 (45.22)	<0.001
Dyslipidaemia	3108 (14.4)	127 (20.22)	<0.001
Chronic kidney disease	844 (3.91)	56 (8.92)	<0.001
Obesity	2613 (12.11)	86 (13.69)	0.23
Gastrectomy type			0.74
Total	3671 (17.01)	110 (17.52)	
Subtotal	17,912 (82.99)	518 (82.48)	

Abbreviations: BMI, body mass index; DBP, diastolic blood pressure; GFR, glomerular filtration rate; HDL, high‐density lipoprotein; LDL, low‐density lipoprotein; SBP, systolic blood pressure; SD, standard deviation; TG, triglyceride.

^a^ Data are expressed as no. (%) of participants unless indicated otherwise. Percentages have been rounded and may not total 100.

^b^ Non‐drinking: alcohol intake 0 g/day, mild drinking: alcohol intake <30 g/day, heavy drinking: alcohol intake ≥30 g/day based on self‐reported questionnaires.

Of the 22,211 patients in the total study population, 2006 (9.0%) were current smokers, 3127 (14.1%) were drinkers (both mild and heavy) and 15,916 (71.7%) were physically inactive, as determined by the post‐surgery lifestyle questionnaire (second exam).

### 3.2 Changes in lifestyle behaviours after surgery

The associations between changes in lifestyle behaviours and the risk of CVD development are presented in Table 2. Regarding smoking, 69.9% (*n* = 15,527) remained non‐smokers, 1.0% (*n* = 221) started smoking, 21.1% (*n* = 4678) quit smoking and 8.0% (*n* = 1785) were persistent smokers. Regarding alcohol intake, 46.8% (*n* = 10,394) remained non‐drinkers, 1.6% (*n* = 366) started drinking, 39.1% (*n* = 8690) quit drinking and 12.4% (*n* = 2761) were persistent drinkers. As for physical activity, 60.1% (*n* = 13,342) remained physically inactive, 17.6% (*n* = 3899) started regular physical activity, 11.6% (*n* = 2574) quit regular physical activity and 10.8% (*n* = 2396) continued regular physical activity.

**TABLE 2:** Risk of cardiovascular disease according to changes in lifestyle behaviours.

	*N*	CVD, *N*	Duration, person‐years	IR per 1000 person‐years	HR (95% CI)		
					Model 1	Model 2	Model 3
Smoking							
Non‐smoking	15,527	416	76,962.50	5.41	1 (Ref.)	1 (Ref.)	1 (Ref.)
New smoking	221	12	1107.41	10.84	2.01 (1.13–3.56)	1.98 (1.11–3.53)	1.85 (1.04–3.30)
Smoking cessation	4678	122	23,993.06	5.08	0.94 (0.77–1.15)	1.16 (0.93–1.43)	1.16 (0.93–1.43)
Persistent smoking	1785	78	8498.68	9.18	1.71 (1.34–2.17)	1.83 (1.43–2.36)	1.72 (1.33–2.22)
Alcohol intake							
Non‐drinking	10,394	297	51,515.23	5.77	1 (Ref.)	1 (Ref.)	1 (Ref.)
New drinking	366	12	1851.31	6.48	1.12 (0.63–2.00)	1.11 (0.62–1.98)	1.02 (0.57–1.83)
Alcohol abstinence	8690	219	43,468.77	5.04	0.88 (0.73–1.04)	1.02 (0.84–1.23)	1.03 (0.85–1.24)
Persistent drinking	2761	100	13,726.34	7.29	1.26 (1.01–1.59)	1.37 (1.08–1.75)	1.23 (0.96–1.58)
Physical activity							
Remain physically inactive	13,342	384	66,068.15	5.81	1 (Ref.)	1 (Ref.)	1 (Ref.)
Start regular physical activity	3899	92	19,753.32	4.66	0.80 (0.64–1.00)	0.86 (0.68–1.08)	0.87 (0.69–1.10)
Quit regular physical activity	2574	82	12,861.85	6.38	1.10 (0.86–1.39)	1.00 (0.79–1.27)	0.98 (0.77–1.24)
Continue regular physical activity	2396	70	11,878.33	5.89	1.01 (0.79–1.31)	0.97 (0.75–1.26)	0.99 (0.76–1.28)

Abbreviations: CI, confidence interval; CVD, cardiovascular disease; HR, hazard ratio; IR, incidence rate.

Model 1: Non‐adjusted;

Model 2: Adjusted for age and sex;

Model 3: Adjusted for age, sex, smoking, alcohol intake, physical activity, diabetes, hypertension, dyslipidaemia, body mass index, glomerular filtration rate.

Most patients who previously smoked or drank, quit smoking (4678/6463 = 72.4%) and abstained from alcohol (8690/11,451 = 75.9%) after surgery. Only a small proportion of patients who were previously physically inactive started regular physical activity (3899/17,241 = 22.6%) and more than half (2574/4970 = 51.8%) of patients who were previously physically active quit regular physical activity after surgery.

### 3.3 Association of lifestyle modifications with the risk of new‐onset CVD

In the multivariable‐adjusted Cox proportional hazards model analysis, both new smoking and persistent smoking were associated with an elevated risk of CVD development with hazard ratios of 1.85 and 1.72, respectively, compared to non‐smoking (Table 2 and Figure S1). Smoking cessation did not demonstrate a significant association with CVD risk when compared to non‐smokers (Hazard ratio (HR): 1.16, 95% confidence interval (CI): 0.93–1.43). Changes in alcohol intake and physical activity status did not show significant associations with CVD risk (Table 2 and Figure S1).

When the CVD outcome was divided into myocardial infarction and stroke, the risk of new‐onset myocardial infarction had the same tendency as the risk of CVD, with elevated risk observed for new smoking (HR: 2.35, 95% CI: 1.15–4.79) and persistent smoking (HR: 1.61, 95% CI: 1.12–2.31), compared to non‐smoking (Table S2). Regarding stroke, the risk of new‐onset stroke was significantly higher for persistent smoking (HR: 1.73, 95% CI: 1.21–2.48), but not for new smoking (HR: 1.19, 95% CI: 0.44–3.21) (Table S3). Changes in alcohol intake and physical activity status did not show significant associations with the risk of either myocardial infarction or stroke (Tables S2 and S3).

### 3.4 Subgroup analyses

Subgroup analyses according to changes in lifestyle behaviours, stratified by age, sex and type of gastrectomy are shown in Tables 3, 4 and Table S4. While persistent smoking was associated with a higher risk of developing CVD compared to non‐smoking in both sexes, the risk was notably pronounced in females, with a 3.89‐fold risk elevation (HR: 3.89, 95% CI: 1.20–12.62) while men exhibited a 1.65‐fold elevated risk (HR: 1.65, 95% CI: 1.27–2.15) (Table 3). For alcohol intake, a sex‐specific effect was noticed. Female patients who began drinking after gastrectomy, exhibited a notably increased risk of developing CVD compared to non‐drinkers, with an HR of 2.89 (95% CI: 1.06–7.88). However, this correlation was absent among male patients (HR: 0.76, 95% CI: 0.37–1.55) (Table 4). In terms of age, young age (<65 years) was associated with an increased risk of CVD in new smokers (HR: 3.11, 95% CI: 1.57–6.18) compared to non‐smokers. Remarkably, this association was not evident among new smokers aged 65 and over (HR: 0.83, 95% CI: 0.26–2.59) (Table 3). No discernible disparities in CVD risk were observed in relation to changes in physical activity, irrespective of age, sex or gastrectomy type (Table S4).

**TABLE 3:** Risk of cardiovascular disease development according to changes in smoking behaviours stratified by age, sex and type of gastrectomy.

Subgroup	Smoking	*N*	CVD, *N*	Duration, person‐years	IR per 1000 person‐years	HR^a^ (95% CI)
Age						
<65	Non‐smoking	9797	143	49,789.55	2.87	1.00 (Ref.)
	New smoking	153	9	785.62	11.46	3.11 (1.57–6.18)
	Smoking cessation	3698	73	19,311.66	3.78	1.17 (0.86–1.58)
	Persistent smoking	1313	41	6337.64	6.47	1.72 (1.19–2.50)
≥65	Non‐smoking	5730	273	27,172.96	10.05	1.00 (Ref.)
	New smoking	68	3	321.79	9.32	0.83 (0.26–2.59)
	Smoking cessation	980	49	4681.4	10.47	1.13 (0.82–1.55)
	Persistent smoking	472	37	2161.04	17.12	1.74 (1.21–2.50)
Sex						
Male	Non‐smoking	8965	283	44,139.65	6.41	1.00 (Ref.)
	New smoking	206	12	1029.82	11.65	1.90 (1.06–3.39)
	Smoking cessation	4479	118	23,023.16	5.13	1.12 (0.89–1.39)
	Persistent smoking	1735	75	8277.06	9.06	1.65 (1.27–2.15)
Female	Non‐smoking	6562	133	32,822.86	4.05	1.00 (Ref.)
	New smoking	15	0	77.59	0.00	–
	Smoking cessation	199	4	969.9	4.12	1.61 (0.59–4.41)
	Persistent smoking	50	3	221.62	13.54	3.89 (1.20–12.62)
Gastrectomy type						
Total	Non‐smoking	2618	68	12,444.93	5.46	1.00 (Ref.)
	New smoking	32	2	166.23	12.03	1.96 (0.47–8.09)
	Smoking cessation	861	21	4262.97	4.93	1.06 (0.63–1.78)
	Persistent smoking	270	19	1213.39	15.66	2.82 (1.63–4.90)
Subtotal	Non‐smoking	12,909	348	64,517.57	5.39	1.00 (Ref.)
	New smoking	189	10	941.17	10.63	1.84 (0.97–3.46)
	Smoking cessation	3817	101	19,730.09	5.12	1.18 (0.93–1.49)
	Persistent smoking	1515	59	7285.29	8.1.	1.53 (1.14–2.05)

Abbreviations: CI, confidence interval; CVD, cardiovascular disease; HR, hazard ratio; IR, incidence rate.

^a^ Adjusted for age, sex, smoking, alcohol intake, physical activity, diabetes, hypertension, dyslipidaemia, body mass index and glomerular filtration rate.

**TABLE 4:** Risk of cardiovascular disease development according to changes in alcohol intake behaviours stratified by age, sex and type of gastrectomy.

Subgroup	Alcohol intake	*N*	CVD, *N*	Duration, person‐years	IR per 1000 person‐years	HR^a^ (95% CI)
Age						
<65	Non‐drinking	6171	92	31,487.9	2.92	1.00 (Ref.)
	New drinking	248	8	1290.18	6.20	1.49 (0.72–3.11)
	Alcohol abstinence	6510	114	33,178.99	3.44	1.03 (0.76–1.38)
	Persistent drinking	2032	52	10,267.39	5.06	1.20 (0.82–1.74)
≥65	Non‐drinking	4223	205	20,027.33	10.24	1.00 (Ref.)
	New drinking	118	4	561.13	7.13	0.62 (0.23–1.69)
	Alcohol abstinence	2180	105	10,289.78	10.20	1.03 (0.80–1.33)
	Persistent drinking	729	48	3458.95	13.88	1.27 (0.90–1.77)
Sex						
Male	Non‐drinking	5042	179	24,666.82	7.26	1.00 (Ref.)
	New drinking	287	8	1471.62	5.44	0.76 (0.37–1.55)
	Alcohol abstinence	7500	203	37,578.01	5.40	0.99 (0.81–1.21)
	Persistent drinking	2556	98	12,753.23	7.68	1.22 (0.94–1.57)
Female	Non‐drinking	5352	118	26,848.41	4.40	1.00 (Ref.)
	New drinking	79	4	379.69	10.53	2.89 (1.06–7.88)
	Alcohol abstinence	1190	16	5890.76	2.72	1.22 (0.71–2.09)
	Persistent drinking	205	2	973.11	2.05	0.92 (0.22–3.84)
Gastrectomy type						
Total	Non‐drinking	1753	48	8317.59	5.77	1.00 (Ref.)
	New drinking	54	1	265.98	3.76	0.43 (0.06–3.19)
	Alcohol abstinence	1600	44	7731.15	5.69	1.17 (0.75–1.83)
	Persistent drinking	374	17	1772.81	9.59	1.42 (0.78–2.59)
Subtotal	Non‐drinking	8641	249	43,197.64	5.76	1.00 (Ref.)
	New drinking	312	11	1585.33	6.94	1.15 (0.63–2.11)
	Alcohol abstinence	7090	175	35,737.62	4.90	1.00 (0.81–1.24)
	Persistent drinking	2387	83	11,953.53	6.94	1.21 (0.92–1.58)

Abbreviations: CI, confidence interval; CVD, cardiovascular disease; HR, hazard ratio; IR, incidence rate.

^a^ Adjusted for age, sex, smoking, alcohol intake, physical activity, diabetes, hypertension, dyslipidaemia, body mass index and glomerular filtration rate.

## 4 DISCUSSION

Cardiovascular disease may compromise the quality of life and influence the long‐term prognosis of cancer patients. However, cardiovascular risk factors are often overlooked during survivorship care.^7^ A previous study reported that nearly one‐third of cancer patients did not engage in discussions about health promotion with their healthcare providers.^7^ Given the impact of CVD on prognosis and survival among cancer patients, our study aims to highlight the need for both medical providers and patients to recognise and address modifiable cardiovascular risk factors and initiate discussions during cancer treatment and follow‐up.

In this study, we analysed the lifestyle behaviours of gastric cancer patients who underwent surgery and explored the associations of lifestyle modifications with new‐onset CVD. Our findings revealed that the majority of patients who did not smoke or drink before surgery maintained their non‐smoking and non‐drinking habits postoperatively. Furthermore, most patients who had been smokers or drinkers before surgery quit smoking and abstained from alcohol after surgery. This appears to reflect physicians' and patients' awareness regarding the potentially adverse prognosis associated with smoking and alcohol intake in cancer patients.^19^, ^20^, ^21^ Interestingly, the majority of those who were physically inactive before surgery remained so afterward and over half of the patients who were physically active before surgery ceased their regular physical activity postoperatively. The diagnosis of gastric cancer and the subsequent surgical intervention might place considerable physical strain on patients, possibly contributing to decreased physical activity levels after surgery.

While new smoking and persistent smoking were associated with an increased risk of CVD, patients who previously smoked but quit after gastrectomy did not demonstrate a significant difference in the risk of developing CVD compared to non‐smokers. This indicates that smoking after surgery significantly increases the risk of CVD, regardless of previous smoking history. It also suggests that quitting smoking can potentially lower the CVD risk to levels similar to non‐smokers. Lee et al. recently assessed CVD risk among cancer survivors based on changes in smoking behaviours. They found that CVD events were most common in persistent smokers, followed by new smokers, those who quit smoking and non‐smokers—a trend that aligns with our study's observations.^14^ The difference in the risk of CVD in smoking cessation between their study and ours may be due to differences in cancer types and stages (their study included 3‐year cancer survivors of all cancer types). Wang et al. evaluated CVD mortality in cancer survivors and reported that persistent smokers had significantly higher CVD mortality compared to non‐smokers, but this was not observed in those who quit smoking, consistent with our results.^13^

Our study found no significant correlation between changes in alcohol intake and CVD development in gastric cancer patients. Previous research on alcohol intake and its association with CVD risk reported inconsistent findings, ranging from U‐ or J‐shaped associations to consistent risk increases for all amounts of drinking.^22^, ^23^ Moreover, the effect of changes in drinking behaviours on CVD risk has not been identified either in the general population or among cancer patients.

Changes in physical activity status did not appear to significantly affect CVD development in gastric cancer patients who underwent surgery except female new drinkers. While previous studies have shown that regular physical activity can help prevent CVD in both the general population and cancer patients, the effect of changes in physical activity (such as starting exercise) on CVD risk remains unclear, especially for gastric cancer patients.^24^, ^25^

While patients who developed CVD were more likely to be male and older, as shown in Table 1, smoking or drinking appeared to have a greater impact on CVD in females and younger patients. Subgroup analyses revealed that female patients who continued smoking or started drinking after surgery were at especially high risk for developing CVD, compared to non‐smokers and non‐drinkers. Previous studies have indicated that smoking and drinking might pose greater CVD risks for females than males.^26^, ^27^, ^28^, ^29^ Although the underlying mechanisms for this disparity are not fully understood, they could be related to differences in smoking and drinking patterns between sexes, the effect of smoking on lowering oestrogen levels (oestrogen has protective effects against atherosclerosis in female), genetic differences related to thrombin signalling, or metabolic differences (females generally have a lower body water content and lower alcohol dehydrogenase activity compared to males, which may contribute to higher CVD risk in females).^26^, ^28^, ^29^, ^30^ Regarding age, a recent study reported that starting smoking at a young age was associated with a higher risk of CVD mortality than starting at an older age, which could explain the high CVD risk in younger new smokers in our study.^31^

When the study outcome was categorised into myocardial infarction and stroke, the correlation between new smoking and stroke was less robust than the association with myocardial infarction. This may reflect the different pathophysiology of the two conditions: while myocardial infarction is predominantly due to atherosclerotic disease, strokes can arise from a variety of causes (cardioembolic, atherosclerotic, lacunar, etc.).^32^

This study has several limitations. We used the NHIS claims data based on ICD‐10‐CM codes and health examination results, which may not accurately reflect patients' medical conditions. Moreover, while we adjusted for numerous confounders, including demographic factors, comorbidities and lifestyle behaviours, we did not account for other potentially impactful variables such as dietary habits (e.g. intake of processed foods, saturated fats and low fibre—all of which are known correlates of CVD risk), cancer stage and medication history. Although cancer stage is known to be a critical factor for prognosis and may have associations with CVD outcomes, the NHIS database does not provide this information, which highlights the need for it to be included in future studies. Furthermore, we identified changes in lifestyle behaviours in a binary fashion. Changes in the lifestyle behaviours that did not lead to a transition between categories were not discernible using this method. Lastly, due to the reliance on epidemiological data, we were unable to delve into the underlying mechanisms linking alterations in lifestyle factors with the development of CVD.

To our knowledge, this is the first study to evaluate the associations between lifestyle modifications and new‐onset CVD in gastric cancer patients. Our findings show that persistent smokers, new smokers and female new drinkers face elevated risks of developing CVD. Notably, while a significant proportion of patients ceased smoking and abstained from alcohol, around a quarter continued to smoke or drink even after gastric cancer surgery, underscoring the need for healthcare providers to advocate for smoking cessation and alcohol abstinence.

## AUTHOR CONTRIBUTIONS

**Bokyung Kim:** Conceptualization (equal); data curation (equal); formal analysis (equal); methodology (equal); resources (equal); visualization (equal); writing – original draft (equal); writing – review and editing (equal). **Kyungdo Han:** Conceptualization (equal); data curation (equal); formal analysis (equal); investigation (equal); methodology (equal); project administration (equal); software (equal); supervision (equal); writing – original draft (equal); writing – review and editing (equal). **Soo‐Jeong Cho:** Conceptualization (equal); data curation (equal); formal analysis (equal); funding acquisition (equal); investigation (equal); methodology (equal); project administration (equal); supervision (equal); writing – original draft (equal); writing – review and editing (equal).

## CONFLICT OF INTEREST STATEMENT

The authors declare no conflicts of interest.

## ETHICS STATEMENT

The authors assert that all procedures contributing to this work comply with the ethical standards of the relevant national and institutional committees on human experimentation and with the Helsinki Declaration of 1975, as revised in 2008. The requirement for written consent was waived by the Institutional Review Board (IRB) of Seoul National University Hospital (No. 2308‐104‐1459).

## Supporting information


Data S1:

## Data Availability

The data that support the findings of this study are available from the National Health Insurance Service (NHIS) of South Korea which can be accessed by visiting the NHIS data center after approval (https://nhiss.nhis.or.kr/bd/ab/bdaba000eng.do).
