# Supplementary material for: Association of lifestyle modification with the development of cardiovascular disease in gastric cancer patients who underwent gastrectomy: A nationwide population‐based study
Source: Cancer Med. 2024 Jul 24;13(14):e70038. doi: 10.1002/cam4.70038 (PMC11267560; doi:10.1002/cam4.70038)
Supplement: Supplementary file 1 — Data S1: [file CAM4-13-e70038-s001.docx]

**Supplemental Material**

Figure S1. Kaplan-Meier curves of cumulative probability of cardiovascular disease (CVD) development stratified by changes in behaviors of smoking, alcohol intake, and physical activity. (A) CVD development according to changes in smoking behaviors. (B) CVD development according to changes in alcohol intake behaviors. (C) CVD development according to changes in regular physical activity.

Table S1. Definitions of covariates

Table S2. Risk of myocardial infarction according to changes of lifestyle behaviors

Table S3. Risk of stroke according to changes of lifestyle behaviors

Table S4. Risk of cardiovascular disease development according to changes in regular physical activity stratified by age, sex, and type of gastrectomy

**Figure S1. Kaplan-Meier curves of cumulative probability of cardiovascular disease (CVD) development stratified by changes in behaviors of smoking, alcohol intake, and physical activity. (A) CVD development according to changes in smoking behaviors. (B) CVD development according to changes in alcohol intake behaviors. (C) CVD development according to changes in regular physical activity.**

**
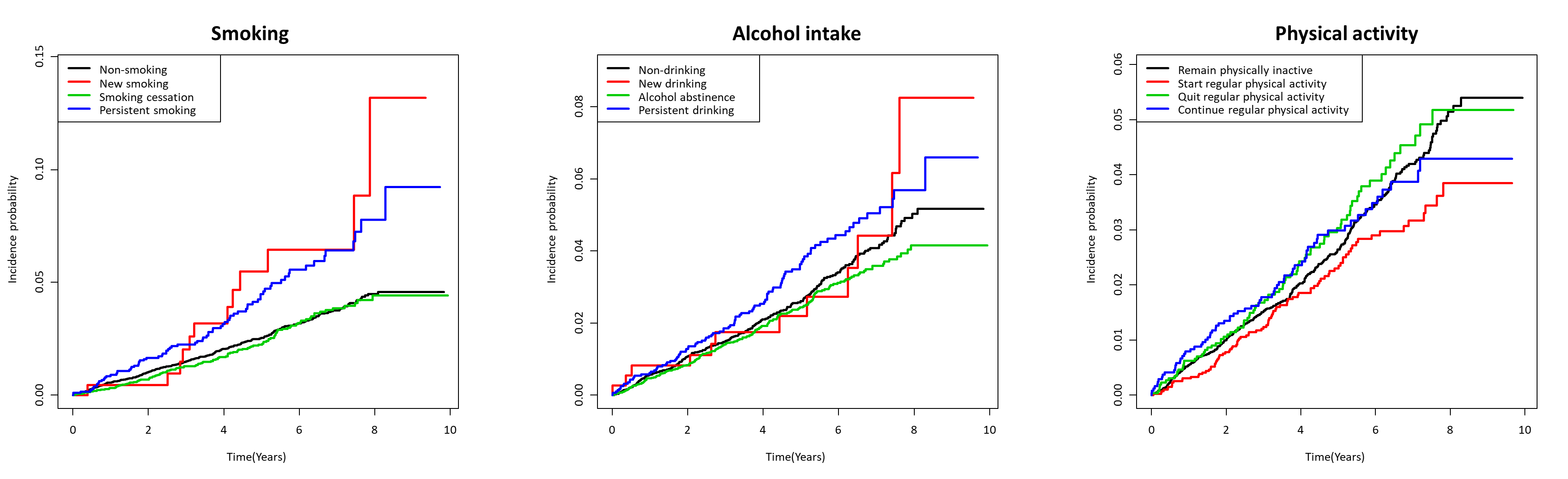
**

**Table S1. Definitions of covariates**

| **Diagnosis** | **ICD-10 codes and definition** | **Diagnostic definition** |
| --- | --- | --- |
| Comorbidities |  |  |
| Diabetes mellitus | E11, E12, E13, E14; and minimum 1 prescription of anti-diabetic drugs (sulfonylureas, metformin, meglitinides, thiazolidinediones, dipeptidyl peptidase-4 inhibitors, α-glucosidase inhibitors, and insulin)  or fasting blood glucose ≥126 mg/dL | Admission≥1 or outpatient department≥1 |
| Hypertension | I10, I11, I12, I13, I15; and minimum 1 prescription of anti-hypertensive drug (thiazide, loop diuretics, aldosterone antagonist, alpha-/beta-blocker, calcium-channel blocker, angiotensin-converting enzyme inhibitor, angiotensin II receptor blocker)  or systolic blood pressure ≥140 mmHg  or diastolic blood pressure ≥90 mmHg | Admission≥1 or outpatient department≥1 |
| Dyslipidemia | E78; and minimum 1 prescription of antihyperlipidemic medications  or total cholesterol ≥240 mg/dL | Admission≥1 or outpatient department≥1 |
| CKD | ^a^eGFR<60ml/min/1.73m^2^ | Health examinations results (2^nd^ exam) |
| Obesity | BMI ≥25 kg/m2 | Health examinations results (2^nd^ exam) |
| Socioeconomic status |  |  |
| Low income | Income lowest 25% and medical aid |  |

CKD, chronic kidney disease; eGFR, estimated glomerular filtration rate; BMI, body mass index

^a^The eGFR was calculated using the MDRD equation: 186.3 × [sCr (mg/dL)]^–1.154^ × [age (years)]^–0.203^ × (0.742 if female)

**Table S2. Risk of myocardial infarction according to changes of lifestyle behaviors**

|  | N | Myocardial infarction, N | Duration, person-years | IR per 1000 person-years | HR (95% CI) | | |
| --- | --- | --- | --- | --- | --- | --- | --- |
|  |  |  |  |  | Model 1 | Model 2 | Model 3 |
| Smoking |  |  |  |  |  |  |  |
| Non-smoking | 15,527 | 215 | 77,436.78 | 2.78 | 1 (Ref.) | 1 (Ref.) | 1 (Ref.) |
| New smoking | 221 | 8 | 1,111.28 | 7.2 | 2.59 (1.28-5.24) | 2.57 (1.27-5.24) | 2.35 (1.15-4.79) |
| Smoking cessation | 4,678 | 74 | 24,118.60 | 3.07 | 1.10 (0.85-1.43) | 1.31 (0.99-1.74) | 1.30 (0.98-1.73) |
| Persistent smoking | 1,785 | 39 | 8,605.06 | 4.53 | 1.64 (1.17-2.31) | 1.73 (1.22-2.47) | 1.61 (1.12-2.31) |
| Alcohol intake |  |  |  |  |  |  |  |
| Non-drinking | 10,394 | 156 | 51,837.70 | 3.01 | 1 (Ref.) | 1 (Ref.) | 1 (Ref.) |
| New drinking | 366 | 8 | 1,857.76 | 4.31 | 1.43 (0.70-2.91) | 1.40 (0.68-2.86) | 1.33 (0.65-2.72) |
| Alcohol abstinence | 8,690 | 120 | 43,726.73 | 2.74 | 0.91 (0.72-1.16) | 1.02 (0.78-1.32) | 1.03 (0.79-1.34) |
| Persistent drinking | 2,761 | 52 | 13,849.54 | 3.75 | 1.25 (0.91-1.71) | 1.31 (0.94-1.83) | 1.20 (0.86-1.70) |
| Physical activity |  |  |  |  |  |  |  |
| Remain physically inactive | 13,342 | 206 | 66,447.52 | 3.1 | 1 (Ref.) | 1 (Ref.) | 1 (Ref.) |
| Start regular physical activity | 3,899 | 48 | 19,887.61 | 2.41 | 0.77 (0.57-1.06) | 0.81 (0.59-1.11) | 0.83 (0.60-1.13) |
| Quit regular physical activity | 2,574 | 43 | 12,968.36 | 3.32 | 1.07 (0.77-1.48) | 0.98 (0.70-1.36) | 0.96 (0.69-1.33) |
| Continue regular physical activity | 2,396 | 39 | 11,968.24 | 3.26 | 1.05 (0.75-1.48) | 1.00 (0.71-1.41) | 1.02 (0.72-1.44) |
| IR: Incidence rate, HR: Hazard ratio, CI: Confidence interval. | | | | | | | |
| Model 1: Non-adjusted; | | | | | | | |
| Model 2: Adjusted for Age and Sex; | | | | | | | |
| Model 3: Adjusted for Age, Sex, Smoking, Alcohol intake, Physical activity, Diabetes, Hypertension, Dyslipidemia, Body mass index, Glomerular filtration rate | | | | | | | |

**Table S3. Risk of stroke according to changes of lifestyle behaviors**

|  | N | Stroke, N | Duration, person-years | IR per 1000 person-years | HR (95% CI) | | |
| --- | --- | --- | --- | --- | --- | --- | --- |
|  |  |  |  |  | Model 1 | Model 2 | Model 3 |
| Smoking |  |  |  |  |  |  |  |
| Non-smoking | 15,527 | 219 | 77,380.10 | 2.83 | 1 (Ref.) | 1 (Ref.) | 1 (Ref.) |
| New smoking | 221 | 4 | 1,122.84 | 3.56 | 1.26 (0.47-3.39) | 1.26 (0.47-3.40) | 1.19 (0.44-3.21) |
| Smoking cessation | 4,678 | 56 | 24,114.97 | 2.32 | 0.82 (0.61-1.10) | 1.07 (0.79-1.46) | 1.08 (0.79-1.47) |
| Persistent smoking | 1,785 | 40 | 8,565.55 | 4.67 | 1.65 (1.18-2.32) | 1.84 (1.30-2.61) | 1.73 (1.21-2.48) |
| Alcohol intake |  |  |  |  |  |  |  |
| Non-drinking | 10,394 | 158 | 51,782.37 | 3.05 | 1 (Ref.) | 1 (Ref.) | 1 (Ref.) |
| New drinking | 366 | 4 | 1,866.52 | 2.14 | 0.70 (0.26-1.90) | 0.71 (0.26-1.91) | 0.64 (0.23-1.73) |
| Alcohol abstinence | 8,690 | 105 | 43,711.04 | 2.4 | 0.79 (0.62-1.01) | 0.96 (0.73-1.26) | 0.96 (0.73-1.26) |
| Persistent drinking | 2,761 | 52 | 13,823.55 | 3.76 | 1.23 (0.90-1.69) | 1.39 (0.99-1.93) | 1.22 (0.87-1.72) |
| Physical activity |  |  |  |  |  |  |  |
| Remain physically inactive | 13,342 | 200 | 66,413.43 | 3.01 | 1 (Ref.) | 1 (Ref.) | 1 (Ref.) |
| Start regular physical activity | 3,899 | 45 | 19,869.35 | 2.26 | 0.75 (0.54-1.04) | 0.83 (0.60-1.15) | 0.84 (0.61-1.16) |
| Quit regular physical activity | 2,574 | 41 | 12,930.30 | 3.17 | 1.05 (0.75-1.47) | 0.95 (0.68-1.34) | 0.92 (0.66-1.29) |
| Continue regular physical activity | 2,396 | 33 | 11,970.40 | 2.76 | 0.92 (0.63-1.32) | 0.89 (0.61-1.29) | 0.89 (0.62-1.30) |
| IR: Incidence rate, HR: Hazard ratio, CI: Confidence interval. | | | | | | | |
| Model 1: Non-adjusted; | | | | | | | |
| Model 2: Adjusted for Age and Sex; | | | | | | | |
| Model 3: Adjusted for Age, Sex, Smoking, Alcohol intake, Physical activity, Diabetes, Hypertension, Dyslipidemia, Body mass index, Glomerular filtration rate | | | | | | | |

**Table S4. Risk of cardiovascular disease development according to changes in regular physical activity stratified by age, sex, and type of gastrectomy**

| Subgroup | Physical activity | N | CVD, N | Duration, person-years | IR per 1000 person-years | HR^a^ (95% CI) |
| --- | --- | --- | --- | --- | --- | --- |
| Age |  |  |  |  |  |  |
| <65 | Remain physically inactive | 8,961 | 155 | 45,466.36 | 3.41 | 1.00 (Ref.) |
|  | Start regular physical activity | 2,808 | 47 | 14,522.03 | 3.24 | 0.95 (0.68-1.32) |
|  | Quit regular physical activity | 1,621 | 36 | 8,233.91 | 4.37 | 1.17 (0.82-1.69) |
|  | Continue regular physical activity | 1,571 | 28 | 8,002.17 | 3.50 | 0.93 (0.62-1.40) |
| ≥65 | Remain physically inactive | 4,381 | 229 | 20,601.79 | 11.12 | 1.00 (Ref.) |
|  | Start regular physical activity | 1,091 | 45 | 5,231.28 | 8.60 | 0.84 (0.61-1.16) |
|  | Quit regular physical activity | 953 | 46 | 4,627.94 | 9.94 | 0.89 (0.65-1.23) |
|  | Continue regular physical activity | 825 | 42 | 3,876.16 | 10.84 | 1.10 (0.78-1.53) |
| Sex |  |  |  |  |  |  |
| Male | Remain physically inactive | 8,811 | 280 | 43,399.35 | 6.45 | 1.00 (Ref.) |
|  | Start regular physical activity | 2,834 | 78 | 14,330.13 | 5.44 | 0.92 (0.71-1.18) |
|  | Quit regular physical activity | 1,837 | 67 | 9,193.32 | 7.29 | 1.00 (0.76-1.30) |
|  | Continue regular physical activity | 1,903 | 63 | 9,546.89 | 6.60 | 1.03 (0.78-1.35) |
| Female | Remain physically inactive | 4,531 | 104 | 22,668.80 | 4.59 | 1.00 (Ref.) |
|  | Start regular physical activity | 1,065 | 14 | 5,423.18 | 2.58 | 0.73 (0.41-1.28) |
|  | Quit regular physical activity | 737 | 15 | 3,668.53 | 4.09 | 0.97 (0.56-1.66) |
|  | Continue regular physical activity | 493 | 7 | 2,331.45 | 3.00 | 0.82 (0.38-1.77) |
| Gastrectomy type |  |  |  |  |  |  |
| Total | Remain physically inactive | 2,232 | 65 | 10,572.75 | 6.15 | 1.00 (Ref.) |
|  | Start regular physical activity | 690 | 11 | 3,404.98 | 3.23 | 0.61 (0.32-1.15) |
|  | Quit regular physical activity | 462 | 18 | 2,229.53 | 8.07 | 1.20 (0.71-2.03) |
|  | Continue regular physical activity | 397 | 16 | 1,880.26 | 8.51 | 1.38 (0.79-2.40) |
| Subtotal | Remain physically inactive | 11,110 | 319 | 55,495.39 | 5.75 | 1.00 (Ref.) |
|  | Start regular physical activity | 3,209 | 81 | 16,348.34 | 4.95 | 0.93 (0.73-1.19) |
|  | Quit regular physical activity | 2,112 | 64 | 10,632.32 | 6.02 | 0.92 (0.71-1.21) |
|  | Continue regular physical activity | 1,999 | 54 | 9,998.08 | 5.40 | 0.91 (0.68-1.21) |
| CVD: Cardiovascular disease, IR: Incidence rate, HR: Hazard ratio, CI: Confidence interval. | | | | | | |
| ^a^ Adjusted for Age, Sex, Smoking, Alcohol intake, Physical activity, Diabetes, Hypertension, Dyslipidemia, Body mass index, Glomerular filtration rate | | | | | | |
